# Study of a sandwich structure of transparent conducting oxide films prepared by electron beam evaporation at room temperature

**DOI:** 10.1186/1556-276X-7-304

**Published:** 2012-06-14

**Authors:** Po Kai Chiu, Wen Hao Cho, Hung Ping Chen, Chien Nan Hsiao, Jer Ren Yang

**Affiliations:** 1 Instrument Technology Research Center, National Applied Research Laboratories, Taipei, Taiwan; 2 Institute of Materials Science and Engineering, National Taiwan University, Taipei, 10617, Taiwan

**Keywords:** ZnO, E-beam, Ion-beam-assisted deposition (IAD), Room temperature, Transparent conducting oxide (TCO)

## Abstract

Transparent conducting ZnO/Ag/ZnO multilayer electrodes having electrical resistance much lower than that of widely used transparent electrodes were prepared by ion-beam-assisted deposition (IAD) under oxygen atmosphere. The optical parameters were optimized by admittance loci analysis to show that the transparent conducting oxide (TCO) film can achieve an average transmittance of 93%. The optimum thickness for high optical transmittance and good electrical conductivity was found to be 11 nm for Ag thin films and 40 nm for ZnO films, based on the admittance diagram. By designing the optical thickness of each ZnO layer and controlling process parameters such as IAD power when fabricating dielectric-metal-dielectric films at room temperature, we can obtain an average transmittance of 90% in the visible region and a bulk resistivity of 5 × 10^−5^ Ω-cm. These values suggest that the transparent ZnO/Ag/ZnO electrodes are suitable for use in dye-sensitized solar cells.

## Background

A dielectric-metal-dielectric (DMD) layer structure is a low-energy film structure. It can effectively decrease the transmitted light in the near-infrared (NIR) region, usually by reflection and without affecting visible-light transmission properties [1]. DMD transparent electrodes, where a thin metal layer is embedded between two dielectric layers, have been used recently [2-6]. Compared to single-layered transparent conducting oxide (TCO) film electrodes, DMD electrodes are thinner [3-6]. They are also more durable than single-layered metal films as the top oxide layer protects the metal layer.

ZnO can be doped with a wide variety of ions to ensure its applicability in several fields. Typical dopants that have been used to produce conducting films of ZnO belong to the group IIIa elements of the periodic table (B, Al, Ga, In). Thin films of doped ZnO have been prepared using many techniques such as sputtering [7-9], metal organic chemical vapor deposition (MOCVD) [10], vapor transport [11], pulsed laser deposition [12], spray pyrolysis, and pyrosol processes.

It is well known that the optical and electrical properties of very thin metal films vary according to their structures [13]. To realize bulk-like properties, metal films should form a continuous structure, although they must be thin to ensure high transmittance. Among metal films, Ag films have the highest transmittance for visible light and good conductivity at room temperature, and they are already being used as transparent conducting electrodes in indium tin oxide (ITO)-based [5] multilayer devices. However, there are few reports related to the preparation of Ag- and ZnO-based multilayers at room temperature for application in low-resistance transparent electrodes. To compensate for such deficiency, we prepared Ag- and ZnO-based multilayers at room temperature using electron beam (e-beam) evaporation with the aid of collocated ion beams.

The thin-film characteristics of e-beam-evaporated films can be enhanced with ion-beam-assisted deposition (IAD). Another significant advantage of this process is that thin films prepared by ion-assisted e-beam evaporation can be used both with and without low-temperature post-deposition annealing.

In this study, the Macleod software was used to design structures with optimal optical properties. We then investigated the influence of preparation process variables on film properties.

### Experimental procedures

ZnO and Ag films were deposited using an e-beam evaporator and an IAD system. The substrates used in these experiments included a 2-in square glass, a 2-in square plastic (PMMA), and a 1-in Si (100) wafer. The glass substrate and the wafer were cleaned in ultrasonic baths of acetone and ethanol sequentially and blow dried with dry N_2_. The background pressure in the deposition chamber was reduced to 2 × 10^−6^ Torr. The substrates were at room temperature before the start of deposition. Samples were obtained by depositing ZnO and Ag by e-beam evaporation with IAD (sample 1) as well as without IAD (sample 2). The working pressure for the deposition of the first layer (ZnO) was maintained at 4 × 10^−4^ Torr O_2_. The working pressure for the deposition of the third layer (ZnO) was maintained at 6 × 10^−6^ Torr without O_2_ in the 0–10 nm thickness range and at 4 × 10^−4^ Torr O_2_ in the 10–40 nm thickness range. The ZnO deposition rate was 0.2 nm/s. The working pressure for the deposition of the second layer (Ag) was maintained at 6 × 10^−6^ Torr without O_2_. The Ag deposition rate was 0.5 nm/s. The ZnO film was simultaneously bombarded by oxygen ions with ion beam energies of 400–500 W. The Ag film was simultaneously bombarded by argon ions with ion beam energies of 400–500 W.

The crystal orientation of the deposited films was examined by X-ray diffraction (XRD) with Cu Ká radiation. Cross-sectional morphology investigation and electron energy loss spectroscopy (EELS) were carried out using high-resolution transmission electron microscopy (HRTEM). The optical transmission spectra of the films were measured with a Lambda950 spectrometer, and their electrical characteristics were evaluated using an HL5500 system.

## Results and discussion

### Film structure

**Figure 1:**
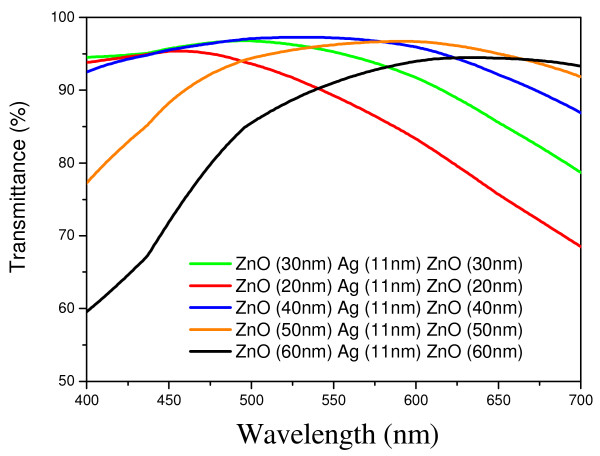
Optical spectra of the substrate/ZnO/Ag/ZnO/air structure simulated by the Macleod software.

A multilayer thin-film structure with the maximum transmittance can be designed using the Macleod software. The admittance diagram of a three-layer ZnO/Ag/ZnO film structure allows determination of the optimal thickness of each layer. The function of the Ag layer was mainly to filter ultraviolet (UV) and infrared (IR) light. The Ag layer should be thicker for better conductivity. On the other hand, ZnO films increase the transmittance of visible light. In reference [5], a 10-mm-thick Ag layer led to fewer variations in sheet resistance, and the transmittance was inversely proportional to the thickness of the metal layer. The optimal thickness of the Ag layer was found to be 11 mm. The thickness of the bottom ZnO layer should be the same as that of the top layers in order to reduce the distance of equivalent admittance (y_E_) and air admittance (y_0_). The minimal reflection condition can be obtained by taking into account these restrictions. In this way, we calculated the value of y_E_ for different thicknesses of ZnO (Table 1). Figure 1 shows the simulation result for the multilayer structure substrate/ZnO/Ag/ZnO/air.

**Table 1:** **Value of y**_**E**_**for different thicknesses of ZnO in the multilayer structure**

**value of y**_**E**_**ZnO/Ag/ZnO**	**Re (Admittance)**	**Im (Admittance)**
20 nm/11 nm/20 nm	1.07	−0.48
30 nm/11 nm/30 nm	1.06	−0.14
40 nm/11 nm/40 nm	1.03	0
50 nm/11 nm/50 nm	1.01	0.3
60 nm/11 nm/60 nm	1	0.67

### Crystallinity

**Figure 2:**
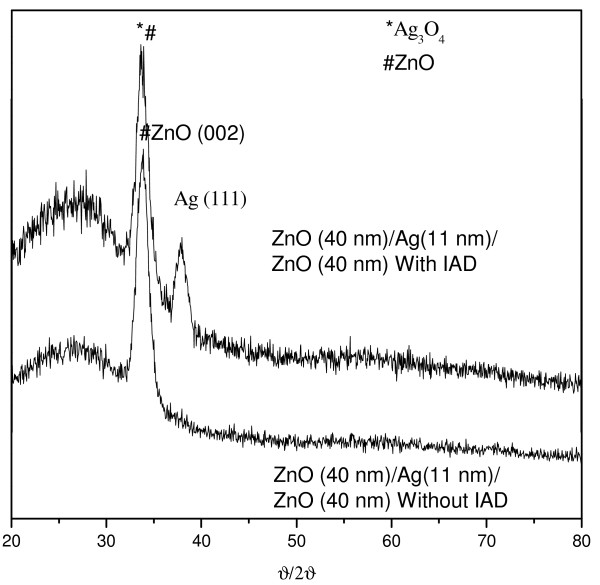
**XRD patterns of the thin-film sandwich structure evaporated on an amorphous substrate and glass by e-beam evaporation.** Sample 1 comprised ZnO/Ag/ZnO structure deposited with IAD and sample 2 comprised the same structure deposited without IAD.

Figure 2 shows the results of the multilayer structure deposited with and without IAD. As seen in the XRD patterns, ZnO thin films evaporated on glass (an amorphous substrate) by e-beam evaporation with IAD preferred to grow in the (002) direction. Moreover, the Ag crystalline peak can clearly be seen in sample 1. This is thought to be the result of using a high momentum ion beam; such beams can increase the evaporation rate and decrease the amount of Ag oxidized.

### Optical properties

**Figure 3:**
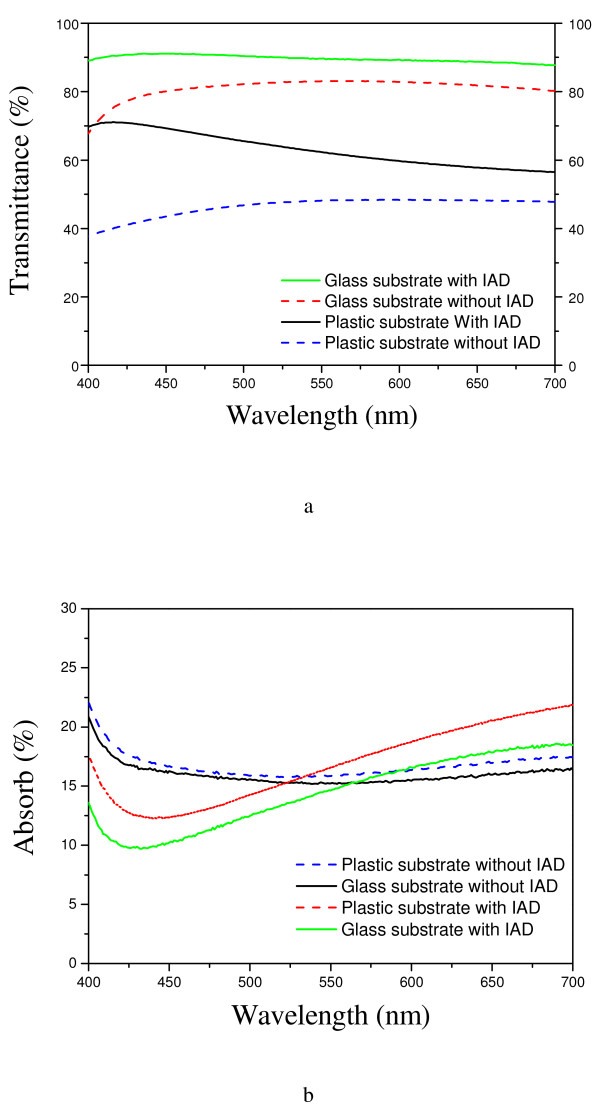
Optical spectra of Ag film deposited using the two different evaporation processes and on two different substrates.

Figure 3a shows the relationship between transmittance and wavelength for the sandwich structure prepared by depositing an Ag layer with and without IAD for the glass and plastic substrates. The ZnO layers were of identical thickness. The transmittance of the single-layer 40-nm ZnO film exceeded 80% over the entire visible light region. The spectral transmittance of the structure including Ag evaporated with IAD was higher than that of the structure including Ag evaporated without IAD. The plastic substrate held water; this caused the oxidation of the metal layer and led to the spectral transmittance being lower than that in case of the glass substrate. In addition, the visible-light optical spectrum of the former was smoother (Figure 3b). Figures 1 and 3 show that the transmittance of the multilayer ZnO/Ag/ZnO film decreased in regions of short and long wavelengths. This may be a result of the absorption being increased because of oxidation of the metal films. The spectral transmittance of an Ag layer deposited with IAD was higher than that of an Ag layer deposited without IAD. In addition, the visible-light optical spectrum of the former was smoother. Thus, IAD was the main process that contributed to the different results in samples 1 and 2.

### Electrical properties

The ideal work function of Ag is 4.4 eV, which is smaller than that of ZnO (5.16-5.3 eV) [13]. When two layers are in contact with each other, the Fermi levels align at equilibrium by the transfer of electrons from Ag to ZnO. The electrical properties would improve under this condition. In this case, there is no barrier for electron flow between Ag and ZnO. Hence, electrons easily transfer from the Ag layer to the ZnO layer. According to Schottky’s theory, we expect high carrier concentrations in multilayer ZnO/Ag/ZnO films. As shown in Table 2, the electrical resistance of the Ag layer prepared without IAD is 10.51 Ω/sq, and this value is more than twice that of the Ag layer prepared with IAD. When the Ag layer is oxidized to Ag_x_O, Ag and O atoms form covalent bonds leading to charge neutrality.

**Table 2:** Electrical behavior of multilayer ZnO/Ag/ZnO samples manufactured with or without IAD

	**Average transmittance** ** (400–700 nm)**	**sheet resistance ** **(ohm/sq)**	**Crystallinity ** **of the metal** **layer**
Glass substrate With IAD	93%	5.08	Polycrystalline
Glass substrate Without IAD	68%	10.51	Amorphous
Plastic substrate With IAD	81%	6.03	Polycrystalline
Plastic substrate Without IAD	46%	13.2	Amorphous

### Microstructure

**Figure 4:**
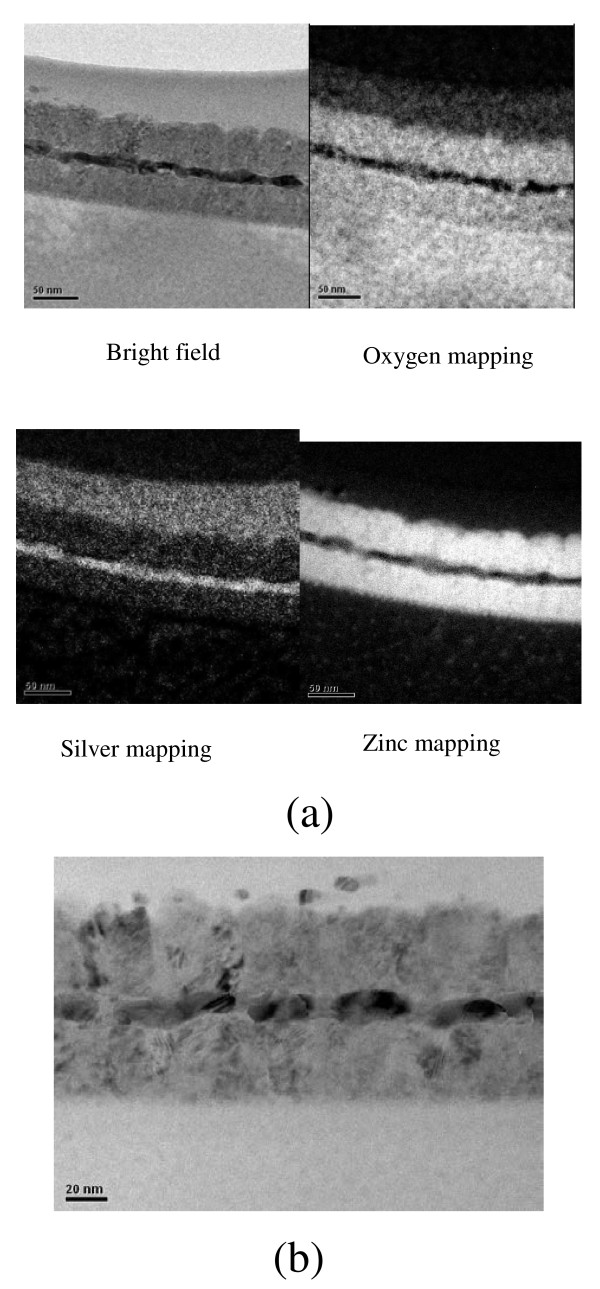
Cross-sectional TEM micrographs of ZnO/Ag/ZnO multilayer structures deposited (a) with IAD (b) without IAD.

Figure 4 shows cross-sectional TEM micrographs of the ZnO/Ag/ZnO multilayer structure manufactured using the IAD process. The film thicknesses measured from TEM micrographs are consistent with the thicknesses suggested by Macleod software simulations. The 11-nm-thick Ag metal layer was a continuous strip that had a nanoscale crystalline structure, and the ZnO films also exhibited polycrystallinity. As seen in the mapping images, the Ag layer did not show the presence of oxides, but diffusion was possible. The Ag metal layer showed discontinuities and formed individual islands (Figure 4b). The formation of partial nanocrystals was also clearly visible. However, irrespective of whether or not the Ag film was continuous, nanocrystals were formed, increasing the sheet resistance of TCO films. Furthermore, as the Ag layer had holes, it would easily lead to oxidation of Ag and reduce the transmittance.

## Conclusion

The effects of the sandwich structure and IAD process on TCO films were investigated. Multilayer films were manufactured using e-beam evaporation at room temperature. The important conclusions of this study are summarized as follows:

1. The thickness and structure of the Ag layer were the main factors that determined the electrical and optical properties of these multilayer structures. Through optical design and simulation, the average transmittance of visible light can be maximized.

2. E-beam evaporation with the IAD process can enhance the optical and crystal properties of ZnO and Ag films. Using this process, it is possible to fabricate flexible TCO films that have an average transmittance of 90% in the visible region and a bulk resistivity of 5 × 10^−5^ Ω-cm.

## Authors’ contributions

PKC: Experimental design and measurements; WHC: Experimental execution; HPC: Experimental execution; CNH: Experimental design and examination of the written report; JRY: Experimental design and examination of the written report. All authors read and approved the final manuscript.

## References

[B1] Tjugum SA Frieling J Johansen GA A compact low energy multibeam gammaray densitometer for pipe-flow measurements Nucl Inst Methods Phys Res B 2002 197 301 309 10.1016/S0168-583X(02)01481-7

[B2] Choi KH Kim JY Lee YS Kim HJ ITO/Ag/ITO multilayer films for the application of a very low resistance transparent electrode Thin Solid Films 1999 341 152 155 10.1016/S0040-6090(98)01556-9

[B3] Kloppel A Kriegseis W Meyer BK Scharmann A Daube C Stollenwerk J Trube J Dependence of the electrical and optical behaviour of ITO-silver-ITO multilayers on the silver properties Thin Solid Films 2000 365 139 146 10.1016/S0040-6090(99)00949-9

[B4] Kloppel A Meyer B Trube J Influence of substrate temperature and sputtering atmosphere on electrical and optical properties of double silver layer systems Thin Solid Films 2001 392 311 314 10.1016/S0040-6090(01)01049-5

[B5] Sawada M Higuchi M Kondo S Saka H Characteristics of indium tin-oxide/silver/indium tin-oxide sandwich films and their application to simple-matrix liquid-crystal displays Jpn J Appl Phys 2001 40 3332 3336 10.1143/JJAP.40.3332

[B6] Jung YS Choi YW Lee HC Lee DW Effects of thermal treatment on the electrical and optical properties of silver-based indium tin oxide/metal/indium tin oxide structures Thin Solid Films 2003 440 278 284 10.1016/S0040-6090(03)00835-6

[B7] Konishi R Noda K Harada H Sasakura H The preparation of transparent ZnO: Al thin films J Crystal Growth 1992 117 939 942 10.1016/0022-0248(92)90888-P

[B8] Martinez MA Herrero J Gutierrez MT Deposition of transparent and conductive Al-doped ZnO thin films for photovoltaic solar cells Solar Energy Mater Solar Cells 1997 45 75 86 10.1016/S0927-0248(96)00066-9

[B9] Roth AP Webb JB Williams D Band-gap narrowing in heavily defect-doped ZnO Phys Rev 1982 12 7836 7839 25

[B10] Wu CC Wu DS Lin PR Chen TN Horng RH Effects of growth conditions on structural properties of ZnO nanostructures on sapphire substrate by metal-organic chemical vapor deposition Nanoscale Research Letters 2009 4 377 384 10.1007/s11671-009-9257-2 20596413 PMC2894013

[B11] Suzuki A Matsushita T Wada N Sakamoto Y Okuda M Transparent conducting Al-doped ZnO thin films prepared by pulsed laser deposition Jpn J Appl Phys 1996 35 L56 L59 10.1143/JJAP.35.L56

[B12] Tiburcio-Silver A Joubert JC Laveau M Études sur la croissance, la structure et la composition de couches minces de ZnO et ZnO dopé a l'indium, obtenues par procédé pyrosol Thin Solid Films 1991 197 195 214 10.1016/0040-6090(91)90232-M

[B13] Parmigiani F Kay E Huang TC Perrin J Jurich M Swalen JD Optical and electrical properties of thin silver films grown under ion bombardment Phys Rev B 1986 33 879 888 10.1103/PhysRevB.33.879 9938347

